# A Comparison Between Lifestyle Interventions and Usual Care Among Indian Adults With Hypertension: A Systematic Review and Meta-Analysis

**DOI:** 10.7759/cureus.109457

**Published:** 2026-05-22

**Authors:** Vincy Nelson, Ayush Mittal, Souvik Manna, Paul T Francis, Nivedita Pavithran

**Affiliations:** 1 Community Medicine, ESIC Medical College and Hospital, Alwar, IND; 2 Research, Amrita Vishwa Vidyapeetham, Kochi, IND; 3 Medicine, ESIC Medical College and Hospital, Alwar, IND; 4 Department of Community Medicine, Amrita Vishwa Vidyapeetham, Kochi, IND

**Keywords:** dash diet, essential hypertension, high blood pressure, high systolic blood pressure, hypertension, lifestyle intervention, mindfulness-based therapy, pharmacological, pre-hypertension, usual care

## Abstract

Cardiovascular diseases (CVDs) rank among the leading causes of global mortality, with high systolic blood pressure (SBP) being a primary driver. In low- and middle-income countries (LMICs) like India, rising hypertension prevalence and limited healthcare access necessitate effective, scalable, and culturally relevant management strategies. This systematic review and meta-analysis (SRMA) aimed to evaluate the clinical effectiveness of various non-pharmacological lifestyle interventions compared to usual care among Indian adults with hypertension or pre-hypertension.

Following the PRISMA (Preferred Reporting Items for Systematic Reviews and Meta-Analyses) guidelines, a comprehensive search of databases, including PubMed, Google Scholar, and Cochrane, was conducted for studies published between January 2000 and December 2025. The inclusion criteria focused on randomized controlled trials (RCTs) and observational studies involving Indian adults (≥18 years) with primary hypertension. Data were synthesized using a narrative review and a random-effects meta-analysis (using R software) for studies with extractable blood pressure outcomes.

Fourteen studies (N = 1,547 participants) met the inclusion criteria, evaluating yoga (e.g., pranayama, meditation), aerobic exercise, dietary salt reduction, and multi-component lifestyle programs. A meta-analysis of seven RCTs revealed that lifestyle interventions significantly reduced SBP by an average of 4.78 mmHg (95% confidence interval (CI): -6.17 to -3.39) and diastolic blood pressure (DBP) by 2.06 mmHg (95% CI: -3.11 to -1.02) compared to usual care. Yoga and meditation-based approaches demonstrated the largest clinical reductions in blood pressure. Statistical heterogeneity was low for SBP (I² = 0%) and moderate for DBP (I² = 16.7%).

Lifestyle interventions, particularly yoga-based and physical activity programs, are effective adjuncts to standard pharmacological therapy for hypertension in India. These low-cost, non-invasive strategies offer significant potential for integration into national non-communicable disease control programs aimed at reducing cardiovascular risk.

## Introduction and background

Cardiovascular diseases (CVDs) account for roughly one-third of fatalities worldwide. High systolic blood pressure (SBP) accounts for 51% of stroke mortality and 45% of ischemic heart disease mortality worldwide [1]. Low- and middle-income countries (LMICs) account for at least three-quarters of all CVD deaths worldwide [2]. Many lifestyle factors are linked to the high prevalence of hypertension, including high-salt and fat-rich diets, insufficient intake of fruits and vegetables, overweight and obesity, unhealthy alcohol consumption, lack of exercise, psychological stress, socioeconomic determinants, and limited access to healthcare [2-4]. People in LMICs frequently lack access to effective and equitable healthcare services [5].

The notion of community control was first established in the World Health Organization's (WHO) cardiovascular program in the latter years of the 1960s, with a particular emphasis on hypertension [6]. The North Karelia Project, a community-based public health intervention begun in 1972 in eastern Finland in response to Finland's high coronary heart disease (CHD) death rate among middle-aged men, pioneered the notion of non-pharmacological therapy. Over 30 years after its inception, the project significantly reduced cardiovascular fatalities through widespread lifestyle and nutritional changes. It successfully demonstrated a drop in mean SBP by 15 and 24 mmHg among men and women, respectively, and mean diastolic blood pressure (DBP) by 8 and 14 mmHg among men and women, respectively [7].

Strong evidence supports the health advantages of decreasing BP through both individual and population-wide interventions [2]. It has been demonstrated that quitting smoking, reducing salt intake, increasing the consumption of fruits and vegetables, exercising frequently, and abstaining from problematic alcohol usage can all lower the risk of hypertension [5]. SBP decreased by 0.32-5.6 mmHg as a result of health promotion and intervention programs that targeted several hypertension risk factors [8]. Numerous studies have demonstrated that a small population-wide drop in SBP (1-2 mmHg) may significantly reduce the incidence of CVD. In 2003, the Joint National Committee, in its seventh report (JNC-7) on the Prevention, Detection, Evaluation, and Treatment of Hypertension, introduced a new term, ‘Pre-hypertension,’ which included SBP ranging from 120 to 139 mmHg and DBP from 80 to 89 mmHg [9]. This new criterion was redefined to highlight the excess risk factors linked to BP in this range and to raise awareness of the significance of preventing hypertension in people of all genders.

The role of low-cost community-based interventions in supporting the self-management of hypertension is particularly important, especially in LMICs. Research on health education interventions from around the globe has shown that suitable and locally applicable strategies enhanced patient awareness and adherence, resulting in modest but beneficial drops in blood pressure. The beneficial effects of these interventions have been reported in many LMICs, including Pakistan, Taiwan, and countries in Africa [10,11]. Non-pharmacological interventions such as physical activity, reducing salt intake, increasing the consumption of fruits and vegetables, and practicing yoga are effective in preventing and controlling hypertension in community-based randomized controlled trials (RCTs) [12-14]. Spiritual well-being can also be viewed as a crucial intervention and is a significant factor in determining both mental and physical health outcomes [15].

There are scarce published systematic reviews on hypertension in Indian adults that specifically focus on individual lifestyle modifications, as well as combination or multi-modality interventions. The current study aims to evaluate the effectiveness of a range of non-pharmacological interventions, including weight reduction, exercise, nutritional modifications (salt and sugar restriction, high-fiber diet, DASH (Dietary Approaches to Stop Hypertension) diet, etc.), alcohol, tea, and coffee restriction, intermittent fasting, stress reduction (through yoga, breathing exercises, mindfulness, etc.), sleep hygiene, smoking cessation, and pollution control (including noise), directly on BP and indirectly on other cardiovascular outcome measures (if measured).

The time frame for observing changes in SBP and DBP was limited to a few weeks in previous studies, which might not have been sufficient to elicit significant changes. Hence, the current review broadens the scope by including studies with longer follow-up periods and also compares the effects of various interventions with usual care to determine the intervention with the greatest effect size among Indian adults. It is well known that BMI categories are different in Asian adults compared to European populations. Hence, studies on non-Asian adults cannot be used to frame guidelines for Indian or South Asian adults. The current systematic review aims to contribute to region-specific guidelines for the prevention and control of hypertension, based on clinical trials and observational analytical studies among Indian adults, especially those comparing non-pharmacological (lifestyle) interventions to usual care or comparing two interventions with each other.

## Review

Review questions

(1) What is the clinical effectiveness of non-pharmacological (lifestyle) interventions in reducing blood pressure among Indian adults with raised blood pressure (hypertension/pre-hypertension)? (2) Which of the non-pharmacological interventions is most effective in reducing blood pressure among the study population?

Methods and analysis

Study Design

This systematic review and meta-analysis (SRMA) was conducted and reported as per PRISMA (Preferred Reporting Items for Systematic Reviews and Meta-Analyses) guidelines [16]. This study was registered on the International Prospective Register of Systematic Reviews (PROSPERO; ID: CRD420261288718).

Search Strategy

Databases such as Cochrane, PubMed, Google Scholar (relevant articles from the first 50 webpages), Embase, and others were searched to retrieve published papers from January 2000 to December 2025. The search terms were identified through an iterative process. The Cochrane Highly Sensitive Search Strategy (sensitivity-maximizing version) was used together with hypertension-related keywords and subject headings to identify randomized trials in Medline. Specific search filters were applied for each database and for RCTs. The searches were conducted in February 2026.

The search strategy was comprehensive, using a combination of controlled vocabulary and free-text terms based on the keywords such as “India”, “Hypertension”, “Lifestyle”, “non-pharmacological”, and “blood-pressure” (Table 1).

**Table 1:** Structured Boolean search framework for relevant literature retrieval

	Search terms
1	Hypertension OR Hypertens* OR High Blood Pressure OR Blood Pressure* OR Pre-hypertension OR Pre-hypertens* OR Pre-hypertension* OR Essential Hypertension OR Lifestyle* OR Non-pharmacological* OR weight* OR exercise* OR DASH* OR nutrition* OR salt* OR sugar* OR sodium* OR potassium* OR fiber* OR alcohol* OR coffee* OR tea* OR fasting* OR Intermittent fasting* OR stress* OR mindfulness* OR yoga* OR pranayama* OR sleep* OR smoking* OR smok* OR pollution* OR indoor air pollution OR air pollution
2	Risk OR associat* OR correlat* OR relation*
3	management OR intervention* OR control* OR comparison* OR usual treatment* OR trial* OR malignan*
4	India OR India* OR Andaman OR Nicobar OR Andhra OR Arunachal OR Assam OR Bihar OR Chandigarh OR Chhattisgarh OR “Dadra and Nagar Haveli” OR Daman OR Diu OR Delhi OR Goa OR Gujarat OR Haryana OR Himachal OR Jammu OR Kashmir OR Jharkhand OR Karnataka OR Kerala OR Lakshadweep OR “Madhya Pradesh” OR Maharashtra OR Manipur OR Meghalaya OR Mizoram OR Nagaland OR Orissa OR Odisha OR Pondicherry OR Punjab OR Rajasthan OR Sikkim OR “Tamil Nadu” OR Telangana OR Tripura OR “Uttar Pradesh” OR Uttarakhand OR “Bengal”
5	#1 AND #2 AND #3 AND #4 6 #5 NOT animal

To find the relevant papers, the titles, abstracts, and full-text articles were systematically searched using these terms. Boolean search operations (OR, AND, NOT) and Medical Subject Headings (MeSH) terms were used to combine the above terms to enhance the effectiveness of the search strategy.

Study Eligibility

All observational studies and RCTs published between January 2000 and December 2025 that explored the effectiveness of non-pharmacological interventions for hypertension were included, either population-based or hospital-based. Studies on secondary hypertension, case reports, or case series, editorials, qualitative studies, commentaries, and conference proceedings were excluded. For selected titles, full-text articles were retrieved. Reference lists of the retrieved articles were searched for additional publications (snowballing). For any further unpublished research, the authors of the retrieved publications were contacted directly.

Inclusion and Exclusion Criteria

The studies were then screened based on study population and study methodology using a set of inclusion and exclusion criteria (Table 2).

**Table 2:** Inclusion and exclusion criteria for study selection

	Inclusion criteria	Exclusion criteria
1	Indian adults aged >18 years	Patient details unavailable
2	Patients with pre-hypertension or primary hypertension	Patients with secondary hypertension
3	Interventions consisting of any non-pharmacological mediation, either singly or in combination with pharmacological treatment	Purely pharmacological or therapeutic intervention without the addition of non-pharmacological interventions
4	Control arm on pure pharmacological treatment (usual care)	Patients with diabetes mellitus, multi-morbidities, or other conditions that make lifestyle interventions less effective

For the classification of hypertension, the American Heart Association and American College of Cardiology (AHA/ACC) guidelines were followed [17]. All non-pharmacological interventions, irrespective of the outcomes or modes of delivery of the intervention, were taken. These included the interventions related to diet (salt reduction, DASH diet, high potassium, high fiber, decreased sugar), physical activity, stress reduction techniques (yoga, meditation, mindfulness), alcohol restriction, coffee and tea intake, sleep hygiene, smoking cessation, and the effect of pollution, etc.

Comparator(s) or Control(s)

Usual care, anti-hypertensive medication, placebo, or other pharmacological or medical interventions, which were compared with non-pharmacological (lifestyle) interventions. The comparator could also be one lifestyle intervention compared with another.

Context

Research only from India was included. Both community-based and hospital-based studies were included in the review. However, only sufficiently homogeneous studies were pooled into the meta-analysis to calculate the pooled estimates. The mean change in systolic and diastolic blood pressure readings following the intervention was the primary outcome.

Data Extraction (Selection and Coding)

Initial eligibility screening was conducted using Rayyan, an automated web-based application for systematic reviews, and subsequently verified by the authors. Abstract and title screening was also performed using Rayyan. Two reviewers independently conducted blinded reviews of the studies (VN and SM), and any disagreements were resolved after discussion with a third assessor (AM). Data extracted by the review team members were cross-checked by other researchers in the team (PF and NP). Information such as the country where the study was conducted, characteristics of the study population (age, sex, and sociodemographic details), sample size, outcome variables, and duration of the intervention was collected. Final values, such as the change in systolic and diastolic blood pressure from baseline, were used to compare the intervention group with the control group or, in non-controlled studies, with baseline values.

Strategy for Data Synthesis

Continuous data were analyzed as mean differences (MD) with 95% confidence intervals (CI), except where studies used different units of measurement. Final values and changes from baseline or from the control group were used to compare the differences resulting from the intervention. Homogeneous studies were synthesized narratively, while RCTs with similar endpoints were synthesized using meta-analysis. An I² value of more than 50% was considered indicative of substantial statistical heterogeneity, and differences in follow-up durations contributed to methodological heterogeneity in the meta-analysis. Clinical heterogeneity was assumed when comparing different lifestyle interventions (diet, yoga, exercise), different age groups, baseline blood pressure, or study population characteristics. A random-effects model was therefore used to generate the overall effect estimates. Rayyan software was used for study screening, while R software was used for meta-analysis. Risk of bias was assessed using the Cochrane RoB-2 tool [18].

A meta-analysis was conducted for seven studies with complete extractable blood pressure outcome data. The mean difference was used as the outcome measure in the analysis. The data were fitted to a random-effects model. The restricted maximum-likelihood estimator was used to determine the degree of heterogeneity, or tau² [19]. The Q-test for heterogeneity (Cochran 1954) and the I² statistic were reported along with the estimate of tau². A prediction interval for the expected results was also provided if any degree of heterogeneity was present (i.e., tau² > 0, independent of the Q-test results). Cook’s distances and studentized residuals were used to assess whether studies were influential or outliers within the model.

Potential outliers were defined as studies with a studentized residual greater than the 100 × (1 - 0.05/(2 × k))th percentile of a standard normal distribution (i.e., applying a Bonferroni correction with two-sided α = 0.05 for k studies included in the meta-analysis). Influential studies were considered those with a Cook’s distance greater than the median plus six times the interquartile range (IQR) of Cook’s distances. Funnel plot asymmetry was examined using the rank correlation test and the regression test, which used the standard error of the observed results as a predictor.

Results

Study Selection and Characteristics

**Figure 1:**
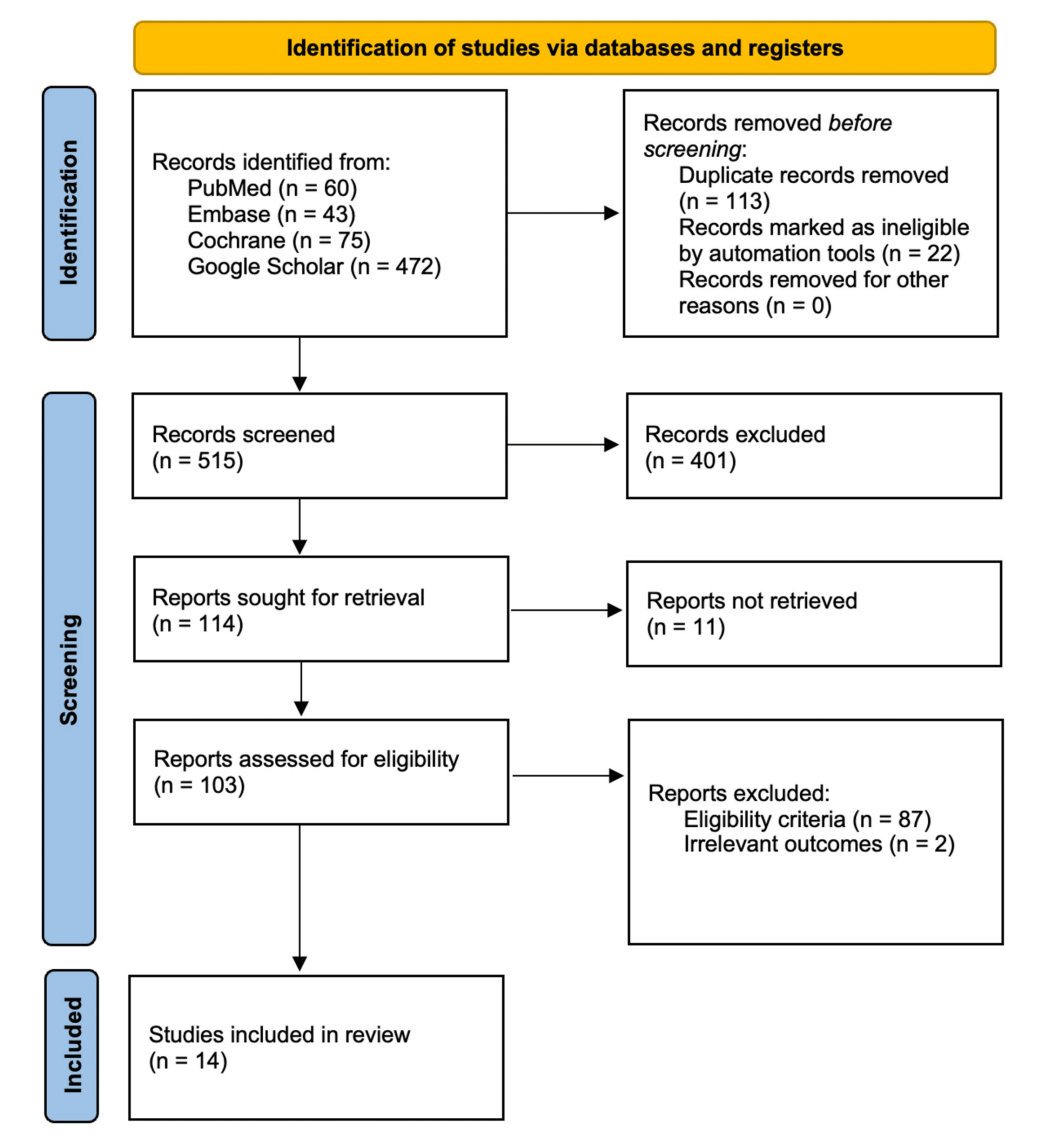
PRISMA flowchart depicting the study selection PRISMA: Preferred Reporting Items for Systematic Reviews and Meta-Analyses

After sequential screening of titles, abstracts, and full-texts, a total of 14 interventional studies conducted among Indian adults with hypertension were included in this SRMA (Figure 1) [20-33].

**Figure 2:**
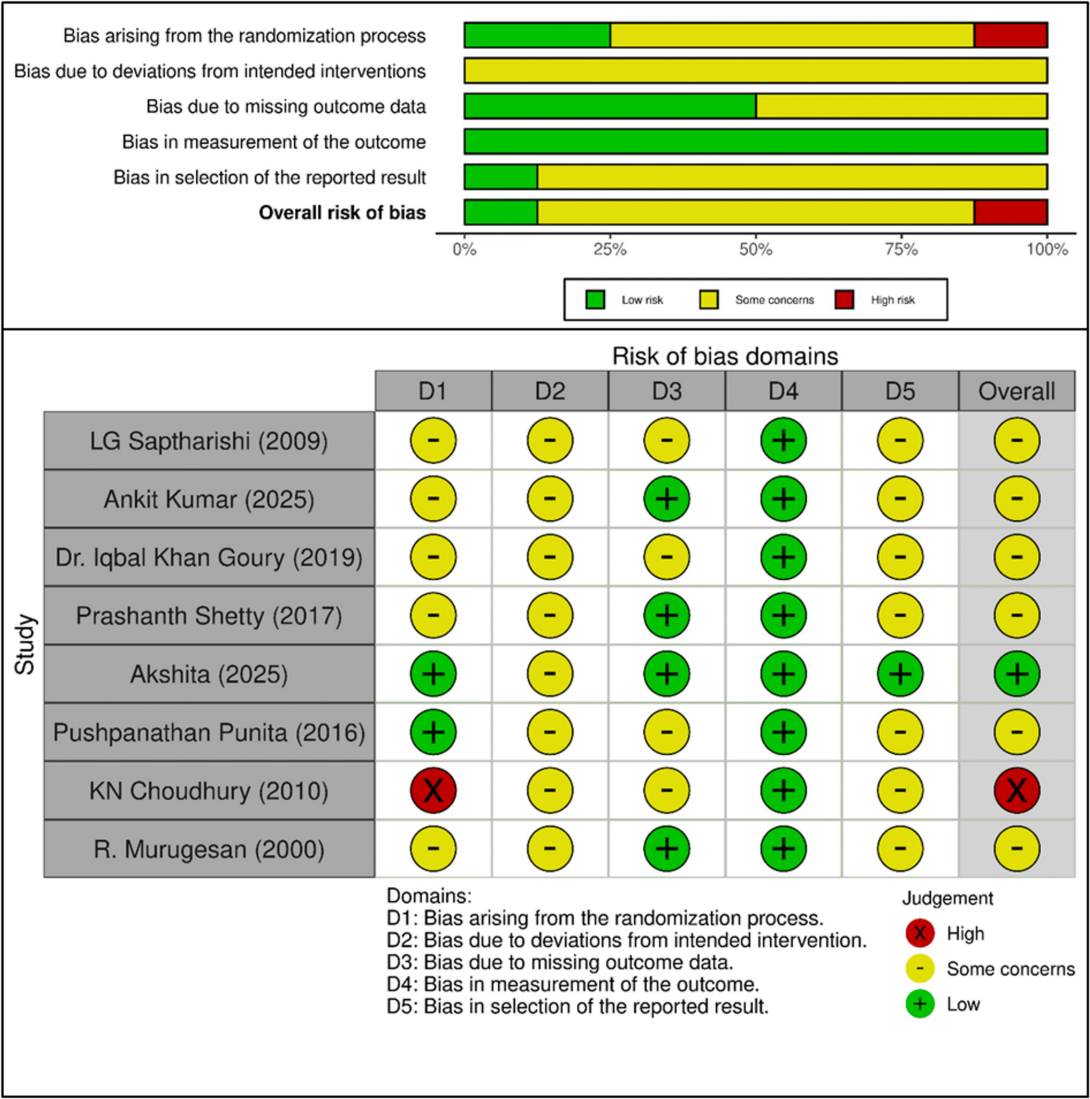
Risk of bias analysis of the eight RCT studies in meta-analysis using the RoB-2 tool (1a and 1b) References: [20,21,24,25,28,29,32,33] RCT: randomized controlled trial

The included studies varied considerably in intervention type, duration, and intensity. Of the 14 studies, three were observational, and 11 were interventional (two non-randomized, one quasi-experimental, and eight randomized clinical trials) (Table 3). These studies were published between 2000 and 2025 and included a total of 1,547 participants (1,009 in the intervention arm and 538 in the control/usual care arm). The studies enrolled adults aged ≥ 18 years, with participants largely diagnosed with primary hypertension (pre-hypertension, stages I and II). Sample sizes ranged from 10 participants per arm to over 217 participants in either arm. Risk of bias was assessed for the eight randomized controlled trials out of the total 14 studies (Figure 2). The interventions evaluated included yoga-based interventions (alternate nostril breathing, yogic lifestyle, meditation), aerobic physical exercise, dietary salt reduction, and combined lifestyle modification programs. Most trials compared lifestyle interventions against usual care or standard control groups.

**Table 3:** Summary of data from the included studies (N = 14) RCT: randomized controlled trial; Quasi: quasi-experimental study; Prospective: prospective observational study; SBP: systolic blood pressure; DBP: diastolic blood pressure; HTN: hypertension

Author	Title	Year	Study design	Setting of research	Population under study	Number of participants in each group	Stage of hypertension	Type of intervention	Findings from the study	Conclusions
Saptharishi et al. [20]	Community-Based Randomized Controlled Trial of Non-pharmacological Interventions in Prevention and Control of Hypertension Among Young Adults	2009	RCT	Community-based	Adults aged > 18 years	Control: 29; physical exercise: 27; salt: 25; yoga: 21	Both pre-hypertension and hypertension stage I	Physical exercise; diet; yoga	SBP reduction: control: 0.2; physical exercise: 5.3; diet: 2.6; yoga: 2.0 | DBP reduction: control: 0.5; physical exercise: 6.0; diet: 3.7; yoga: 2.6	This study showed that while yoga and reducing salt intake were equally effective as non-pharmacological therapies for the prevention and control of HTN in young adults, regular physical activity was more effective than the other two interventions (when taken separately)
Kumar et al. [21]	Comparing the Effectiveness of Aerobic Training, Isometric Handgrip Exercise, and Yoga Therapy to Manage Primary Hypertension: A Randomized Controlled Trial	2025	RCT	Hospital-based	Adults aged > 18 years	Control: 10; physical exercise: 10; yoga: 10	Primary hypertension	Physical exercise; yoga	SBP reduction: control: 1.0; physical exercise: 6.0; yoga: 1.5 | DBP reduction: Control: 1.0; physical exercise: 7.5; yoga: 4.5	Isometric handgrip exercises and yoga treatment are less successful in controlling primary HTN, specifically both SBP and DBP, than aerobic training combined with medication
Khalid and Srivastava [22]	Effect of Alternate Nostril Breathing on Blood Pressure Among Hypertensives in a Selected Urban Community of Gurugram (Haryana)	2024	Quasi	Community-based	Adults aged > 30 years	Control: 30; yoga: 30	Hypertension stage I	Yoga	SBP reduction: control: 1.5; yoga: 7.5 | DBP reduction: control: 1.7; yoga: 7.0	Hypertensives can effectively lower their systolic and diastolic blood pressure by performing alternate breathing exercises twice a day for 10 minutes each time
Hadaye et al. [23]	Effect of Yoga Intervention in the Management of Hypertension: A Preventive Trial	2021	Non-RCT	Hospital-based	Adults aged > 18 years	Control: 72; yoga: 73	Hypertension stage I	Yoga	SBP reduction: control: 3.8; yoga: 7.0 | DBP reduction: control: 2.4; yoga: 5.3	Yoga, along with its three fundamental components - postures, meditation, and breathing - has a small but significant impact on lowering blood pressure
Goury and Khangarot [24]	Reversing Global Burden of Hypertension Through Preksha Meditation and Yogic Lifestyle	2019	RCT	Hospital-based	Adults aged 30-60 years	Control: 30; yoga: 30	Hypertension stage I	Yoga	SBP reduction: control: 1.0; yoga: 31.26 | DBP reduction: control: 0.2; yoga: 12.34	Preksha meditation and a yogic lifestyle are highly effective in treating HTN
Shetty et al. [25]	Effects of Sheetali and Sheetkari Pranayamas on Blood Pressure and Autonomic Function in Hypertensive Patients	2017	RCT	Hospital-based	Adults aged 25-65 years	Control: 30; yoga: 30	Both pre-hypertension and hypertension stage I	Yoga	SBP reduction: control: 0.7; yoga: 16.2 | DBP reduction: not significant	Sheetali and Sheetkaripranayamas reduced SBP in adults of southern Indian descent with HTN
Ali and Sasidharan [26]	Impact of Diet and Lifestyle Modification and Weight Reduction on Essential Hypertension	2022	Prospective	Hospital-based	Adults aged > 20 years	60	Primary hypertension	Diet	SBP reduction: 16.8 | DBP reduction: 8.1	Weight reduction with diet and lifestyle is the definitive therapy for essential HTN
Priya et al. [27]	Impact of Yoga on Blood Pressure and Quality of Life in Patients with Hypertension	2017	Non-RCT	Hospital-based	Adults aged 20-80 years	Control: 27; yoga: 56	Hypertension stage I	Yoga	SBP reduction: control: 1.9; yoga: 6.8 | DBP reduction: control: +0.8; yoga: 4.4	In addition to medical care, basic yoga exercises may be helpful as an additional BP management therapy
Akshita et al. [28]	Effect of Home-based Mobile Guided Pranayam and Yog Nidra Meditation on Blood Pressure and Sleep Quality of Elderly Hypertensive Individuals: A Randomized Controlled Trial	2025	RCT	Hospital-based	Elders aged 60-75 years	Control: 52; yoga: 53	Primary hypertension	Yoga	SBP reduction: control: 6.0; yoga: 10.72 | DBP reduction: control: 1.88; yoga: 2.38	Mobile-guided pranayam and yognidra meditation for the control of hypertension in elderly subjects is effective
Punita et al. [29]	Randomized Controlled Trial of 12-Week Yoga Therapy as a Lifestyle Intervention in Patients With Essential Hypertension and Cardiac Autonomic Function Tests	2016	RCT	Hospital-based	Adults aged 35-55 years	Control: 30; yoga: 25	Hypertension stage I	Yoga	SBP reduction: control: 0.5; yoga: 6.24 | DBP reduction: control: 1.33; yoga: 3.6	Twelve weeks of yoga therapy reduced both SBP and DBP in subjects with HTN
Kumar Panda and Kumar Padhy [30]	Prospective Evaluation of Lifestyle Modification Programs in Reducing Hypertension Among Urban Adults	2025	Prospective	Hospital-based	Adults aged > 18 years	200	Hypertension stage I	Diet + physical activity	SBP reduction: 11.6 | DBP reduction: 6.5	Standardized lifestyle program integrated into routine tertiary outpatient care produced clinically meaningful BP reductions
Gayathri et al. [31]	An Observational Study on Long-Term Outcomes of Lifestyle Interventions in Hypertensive Patients	2024	Prospective	Hospital-based	Adults aged 45-75 years	120	Primary hypertension	Diet; physical activity	SBP reduction: physical exercise: 15; diet: 13 | DBP reduction: physical exercise: 8; Diet: 7	For those with HTN, dietary modifications and consistent aerobic activity successfully reduce blood pressure and improve general health
Choudhury et al. [32]	A Trial on the Effects of Lifestyle Interventions in High-Normal Blood Pressure	2010	RCT	Hospital-based	Adults aged 30-50 years	Control: 217; intervention: 217	Pre-hypertension	Diet + physical activity	SBP reduction: control: 1.1; intervention: 4.1 | DBP reduction: control: 1.2; intervention: 3.5	It was found that non-pharmacological interventions (lifestyle intervention) reduced the mean high normal blood pressure
Murugesan et al. [33]	Effect of Selected Yogic Practices on the Management of Hypertension	2000	RCT	Hospital-based	Adults aged 35-65 years	Control: 11; drug: 11; yoga: 11	Primary hypertension	Drug only; yoga only	SBP reduction: control: 4.19; drug: 23.76; yoga: 33.36 | DBP reduction: control: 1.99; drug: 9.91; yoga: 26.27	Hypertensives benefited from both medication treatment and yoga intervention, but yoga intervention was more successful

Types of Lifestyle Interventions

The interventions studied in these studies were yoga-based and mind-body interventions, yogic lifestyle modification programs, pranayama (alternate nostril breathing), meditation-based approaches (e.g., Preksha meditation, Sheetali and Sheetkari pranayamas, yog nidra, etc), physical activity interventions (structured aerobic exercise training, walking-based programs, etc), dietary and behavioural modification, salt restriction interventions, lifestyle counseling with diet and exercise components, and multicomponent lifestyle packages (combined interventions incorporating yoga, stress reduction, and dietary modification). Most trials compared these interventions with usual care, consisting of standard anti-hypertensive management, brief counseling, or no structured lifestyle program.

Effects on Systolic Blood Pressure (SBP)

Across the 14 studies, lifestyle interventions consistently resulted in greater reductions in systolic blood pressure compared with control groups. Several yoga-based trials demonstrated clinically meaningful SBP reductions, particularly those incorporating meditation and breathing regulation. For example, alternate nostril breathing interventions produced significant improvements in SBP compared with minimal change under usual-care-only conditions [16]. Physical activity interventions, especially aerobic training, showed reductions in SBP, supporting the established role of aerobic exercise in lowering peripheral resistance and improving endothelial function, compared to isometric hand-grip and yoga [17]. Overall, the magnitude of SBP reduction across interventions ranged approximately between 5 and 15 mmHg, depending on intervention type and duration. Such reductions are clinically important, as even modest SBP lowering is associated with substantial reductions in cardiovascular morbidity and or mortality [34].

A sensitivity analysis was conducted by excluding the study by Murugesan et al., which showed an unusually large reduction in systolic blood pressure (SBP). Following this exclusion, the pooled mean difference remained significant, and heterogeneity decreased substantially (I² reduced from 48.26% to 0.00%), indicating that the overall effect was robust. Thus, a total of seven studies were included in the analysis, out of the eight RCTs considered for the meta-analysis.

**Figure 3:**
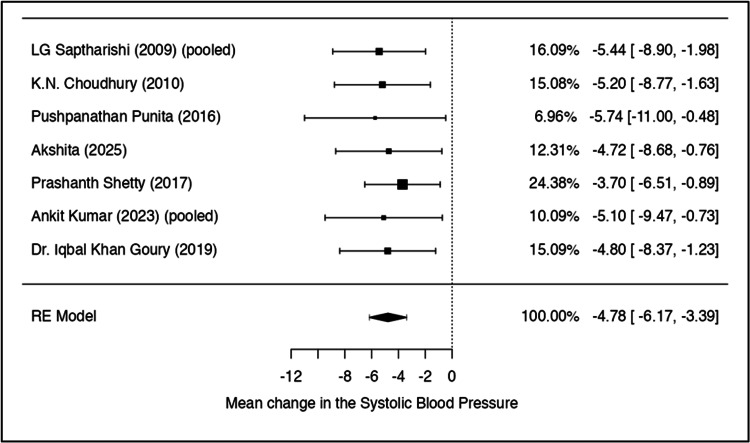
Meta-analysis forest plot showing mean change in SBP due to non-pharmacological interventions (N = 7) The true outcomes (Q(6) = 0.9091, p = 0.9888, tau² = 0.0000, I² = 0.0000%) and outliers show no discernible heterogeneity. Funnel plot asymmetry was not detected by either the regression test or the rank correlation (p = 0.5619 and p = 0.4810, respectively) References: [21,24,25,28,29,32,33] SBP: systolic blood pressure

The forest plot showed that all seven studies demonstrated a significant reduction in SBP with non-pharmacological interventions, with study weights ranging from approximately 6.96% to 24.38%. The largest weight was for the Shetty et al. (2017) study, likely due to a larger sample size or smaller variance. Studies with smaller weights (e.g., Punita et al., 2016) contributed less because of greater uncertainty. Overall, the pooled effect under the random-effects model was -4.78 mmHg (95% CI: -6.17 to -3.39), indicating that lifestyle interventions significantly reduce SBP by an average of 4.8 mmHg compared with usual care. This reduction is clinically meaningful, as a reduction of approximately 5 mmHg SBP can lower the risk of cardiovascular disease (Figure 3).

Publication bias was assessed using Rosenthal’s fail-safe N, Egger’s regression, Begg’s rank correlation, and trim-and-fill methods. The fail-safe N was 112, indicating that a substantial number of unpublished null studies would be required to negate the observed effect. Begg’s (p = 0.562) and Egger’s tests (p = 0.481) were non-significant, suggesting no strong evidence of publication bias. Trim-and-fill analysis suggested three potentially missing studies; however, overall findings remained robust. The pooled SBP reduction of −4.78 mmHg observed in the forest plot is unlikely to be explained by publication bias, and lifestyle interventions appear genuinely effective in lowering systolic blood pressure among Indian adults with hypertension.

Effects on Diastolic Blood Pressure (DBP)

**Figure 4:**
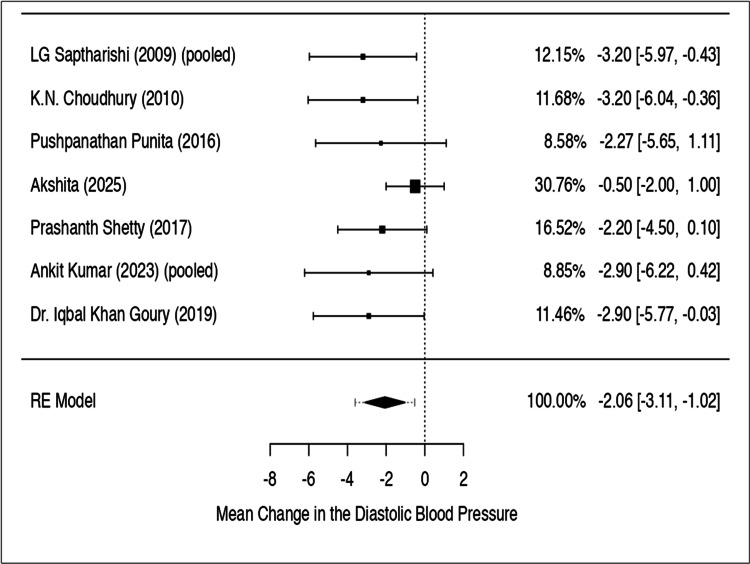
Meta-analysis forest plot showing mean change in DBP due to non-pharmacological interventions (N = 7) The true outcomes show no significant heterogeneity (Q(6) = 5.9184, p = 0.4324, tau² = 0.3345, I² = 16.7249%). A 95% prediction interval for the true outcomes is given by -3.6044 to -0.5227. One study (Akshita et al., 2025) may be considered overly influential based on Cook's distances. Funnel plot asymmetry was detected by the regression test (p = 0.0274), but not by the rank correlation test (p = 1.0000) References: [21,24,25,28,29,32,33] DBP: diastolic blood pressure

DBP outcomes followed similar trends. Most studies reported greater DBP reductions in lifestyle intervention arms compared to controls. Yoga and meditation interventions were particularly effective, likely through autonomic modulation and stress reduction mechanisms. Physical activity and dietary modification trials also demonstrated modest but favorable DBP improvements. A total of seven studies were included in the meta-analysis. The observed mean differences in DBP ranged from -3.2000 to -0.5000 mmHg, with the majority of estimates being negative. The estimated average mean difference based on the random-effects model was û = -2.0635 (95% CI: -3.1071 to -1.0199). Therefore, the average outcome differed significantly from zero (z = -3.8755, p = 0.0001). Although the pooled reduction in DBP was modest, even small decreases in DBP have been associated with meaningful reductions in cardiovascular risk at the population level. Therefore, the observed effect may still be clinically relevant, particularly when considered alongside the significant SBP reduction (Figure 4).

**Figure 5:**
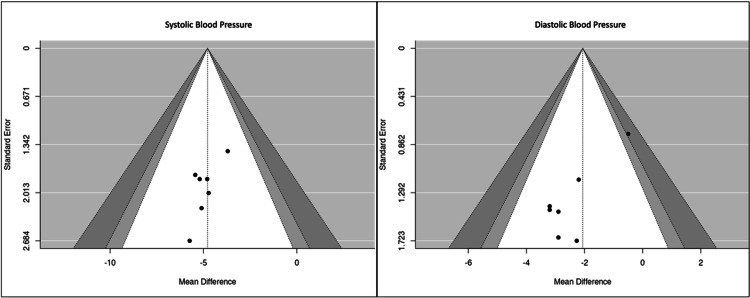
Funnel plots assessing publication bias for SBP (left) and DBP (right) outcomes following lifestyle interventions among Indian adults with hypertension Each point represents an individual study. The vertical dashed line denotes the pooled mean difference, and the sloping dashed lines indicate pseudo 95% confidence limits. Visual inspection demonstrates approximate symmetry, suggesting a low likelihood of publication bias SBP: systolic blood pressure; DBP: diastolic blood pressure

The fail-safe N of 47 suggests that a moderate number of unpublished null studies would be required to negate the observed pooled effect. Begg’s rank correlation test did not indicate asymmetry (p = 1.000); however, Egger’s regression test detected significant funnel plot asymmetry (p = 0.027), suggesting the presence of small-study effects or potential publication bias. Trim-and-fill analysis estimated that four studies may be missing. Additionally, Cook’s distance identified Akshita et al.'s study (2025) as an overly influential study, which may have contributed to the observed asymmetry. Therefore, the pooled findings should be interpreted with caution (Figure 5).

Pooled Results

The pooled results demonstrated that lifestyle interventions significantly reduced both SBP and DBP compared with usual care. However, statistical heterogeneity was considerable (0% for SBP, 16% for DBP), reflecting differences in intervention modalities (yoga vs. diet vs. exercise), duration of follow-up, baseline BP severity, and trial design quality. Due to this heterogeneity, a random-effects model was considered appropriate. In summary, among Indian adults with hypertension, lifestyle interventions consistently improved BP outcomes compared with usual care; yoga and meditation-based approaches produced the largest reductions, while exercise and dietary salt restriction also contributed meaningful benefits. Evidence supports integrating culturally acceptable lifestyle therapies into hypertension management strategies.

Discussion

This systematic review indicates that lifestyle interventions - particularly yoga, aerobic exercise, dietary modification, and meditation - are associated with clinically significant reductions in blood pressure among Indian adults with hypertension. The observed reductions are important because even a 5 mmHg decrease in SBP is linked to major reductions in stroke and coronary mortality globally [35]. Of all the interventions, yoga was the most frequently studied one in the included trials. Evidence suggests that yoga reduces blood pressure through reduced sympathetic activity, improved baroreflex sensitivity, stress reduction, and cortisol modulation. These mechanisms are supported by systematic evidence showing yoga can produce modest but meaningful BP reductions and is an effective adjunctive therapy for hypertension [36].

Another intervention is alternate nostril breathing, which highlights the role of pranayama in modulating autonomic balance, consistent with findings that controlled breathing improves vagal tone [37]. Such breathing-based interventions, including “anuloma-viloma”, may improve baroreflex sensitivity and vagal activity, thereby lowering blood pressure (BP), as reported in some studies [38]. Similarly, mindfulness interventions produce BP improvements, supporting meta-analytic evidence that mindfulness and meditation contribute to hypertension control [39]. The effects of meditation and mindfulness on reducing intraocular pressure, which some studies suggest correlates positively with SBP and DBP, have also been demonstrated in RCTs [40-44].

Two studies in the current SRMA showed that, apart from yoga and mindfulness, aerobic exercise remains a cornerstone of hypertension management [20,21]. Exercise improves endothelial function and reduces peripheral resistance, consistent with global recommendations [45]. Dietary salt reduction also demonstrated benefit, aligning with strong evidence that sodium restriction lowers BP across populations, including South Asians [46].

Hypertension prevalence in India is rising rapidly, with poor control rates and high cardiovascular morbidity [47]. Lifestyle interventions offer scalable, culturally acceptable approaches, especially yoga, which has deep roots in Indian practice. Given limited healthcare access in many regions, community-based interventions may be particularly impactful.

Strengths and limitations

The strengths of the current SRMA lie in its focus on Indian adult hypertensive populations, inclusion of RCT evidence, and consistent direction of benefit across interventions. However, the present review has certain limitations. Several included studies had relatively small sample sizes and variable methodological quality, which may affect the robustness and generalizability of the findings. The inclusion of a few quasi-experimental and non-randomized studies further increases the potential risk of bias. Additionally, heterogeneity in intervention duration, intensity, and implementation protocols limited direct comparability across studies. Variations in study design, including the inclusion of both randomized and non-randomized studies without extensive stratified analysis, may also have contributed to methodological heterogeneity. Furthermore, limited reporting of long-term cardiovascular outcomes restricts the ability to assess the sustained effectiveness of the interventions over time.

Future directions

Future trials should adopt standardized BP measurement protocols and longer follow-up. Lifestyle modification should be emphasized as an adjunct to pharmacotherapy in Indian hypertension care. Yoga-based interventions may offer low-cost and feasible strategies within national programs targeting non-communicable diseases, alongside other interventions. Further high-quality multicentric RCTs are needed to establish optimal intervention packages for Indian settings.

## Conclusions

This SRMA of 14 Indian studies demonstrates that lifestyle interventions provide significant blood pressure reductions compared with usual care. The findings reinforce the importance of non-pharmacological approaches as adjuncts to anti-hypertensive therapy, particularly in resource-constrained settings.
